# Medium-chain Acyl-CoA dehydrogenase deficiency presenting with neonatal pulmonary haemorrhage

**DOI:** 10.1186/s40748-015-0010-9

**Published:** 2015-03-18

**Authors:** Willem Staels, James D’Haese, Els Sercu, Linda De Meirleir, Johan Colpaert, Luc Cornette

**Affiliations:** AZ Sint Jan Brugge-Oostende AV, Neonatal Intensive Care Unit, Bruges, Belgium; Department of Paediatrics and Genetics, Division of Paediatric Endocrinology, Ghent University Hospital, Ghent, Belgium; Department of Paediatrics, Jan Yperman Hospital, Ypres, Belgium; Department of Paediatrics, Division of Paediatric Neurology and Metabolic Diseases, Universtair Ziekenhuis Brussel, Brussels, Belgium; Department of Paediatrics, AZ Groeninge, Kortrijk, Belgium

**Keywords:** Pulmonary haemorrhage, Medium-chain Acyl-CoA Dehydrogenase Deficiency (#MIM 201450)

## Abstract

**Background:**

Medium-chain Acyl-CoA dehydrogenase deficiency (MCADD) is the most common inherited disorder of fatty acid beta-oxidation. Signs and symptoms of MCADD typically appear during infancy or early childhood and include vomiting, lethargy, and hypoglycemia. Pulmonary haemorrhage has previously been described in patients with MCADD, but has always been considered a pre-terminal complication caused by heart failure.

**Case presentation:**

We report on a newborn term infant that presented on the second day of life with signs of encephalopathy, followed by hypovolemia and respiratory distress caused by a severe pulmonary haemorrhage. Fluid resuscitation and mechanical ventilation were initiated and the coagulopathy was corrected by the administration of fresh frozen plasma. Echocardiography revealed a normal cardiac function. After 6 days of full intensive care, the patient survived without sequellae. The clinical presentation in absence of signs of infection raised a strong suspicion for a metabolic disorder and genetic testing revealed MCADD due to a homozygous A985G mutation.

**Conclusion:**

The key towards successful management of severe pulmonary haemorrhage in newborns with a coagulopathy and suspicion of an underlying metabolic disorder consists of adequate mechanical ventilation and aggressive use of fresh frozen plasma, while treating the metabolic decompensation and initiating an early diagnostic work-up. MCADD can lead to acute decompensation and present with complications such as pulmonary haemorrhage independent of cardiac function. Hence, in the context of MCADD, pulmonary haemorrhage should not be considered a pre-terminal complication caused by heart failure, and rather than withdrawing care, intensive treatment must be initiated.

## Background

Clinically significant pulmonary haemorrhage in newborns is rare [1]. In term infants, it is usually associated with meconium aspiration, hypotension or resuscitation with positive pressure ventilation, but its exact pathogenesis remains unknown [1]. Medium-chain Acyl-CoA dehydrogenase deficiency (MCADD) is a rare metabolic disorder in which patients have problems breaking down fatty acids for energy [2,3]. In a typical clinical scenario, a previously healthy child with MCADD presents between the ages three and 24 months with low blood sugar levels, vomiting, and lethargy [3]. Acute episodes are generally triggered by a common illness or by prolonged fasting and liver disease is often seen at presentation [3]. The prognosis of MCADD is excellent once the diagnosis is established and frequent feedings to avoid fasting are instituted [3].

We present a patient with MCADD who presented with rapidly progressive respiratory symptoms due to a massive pulmonary haemorrhage. This is, to the best of our knowledge, the second reported surviving infant with this presentation. In one previous report, pulmonary haemorrhage was suggested to constitute a pre-terminal complication caused by heart failure [4]. However, we documented normal cardiac function and therefore suggest that, in this patient pulmonary haemorrhage was spontaneous and related to the coagulopathy caused by metabolic decompensation.

## Case presentation

The patient was born after an uncomplicated term pregnancy. He weighed 3290 g, appeared vigorous and had perfect Apgar scores. The amniotic fluid was clear. Both parents were healthy, non-consanguineous and there were no siblings. There was no maternal drug abuse. On the second day of life, he presented with poor feeding and grunting. He was lethargic but still reactive upon stimulation and was hypothermic (33°C). His skin was pale, with a prolonged capillary refill time (3 sec) and he had weak femoral pulses. His clinical condition indicated a serious illness with signs of hypovolemic shock. A bolus of normal saline was administered and broad-spectrum antibiotics were initiated. Complete blood count and C-reactive protein were normal, but he had a mild acidosis (pH 7.26) and low blood glucose levels (24 mg/dl). After a bolus of 10% dextrose was given, followed by a continuous infusion glucose levels rose to 72 mg/dl. The patient was referred to our neonatal intensive care unit (NICU).

Upon arrival of the NICU transport team (i.e. 45 minutes after initial admission), a stuporous but arousable patient was seen. The boy was pale and poorly perfused, had tachycardia but still a normal blood pressure. He was grunting, had subcostal retractions, tachypnea and bilateral crackles on auscultation. Another fluid bolus was given and the infant was intubated using rapid sequence intubation. Upon intubation of the trachea, clear red blood returned through the tube. Endotracheal suctioning cleared a large quantity of blood after which mechanical ventilation was initiated. There were no maternal risk factors for vitamin K deficiency and prophylaxis with 2 milligram oral phylloquinone (Vitamin K1) had been given directly after birth.

**Figure 1:**
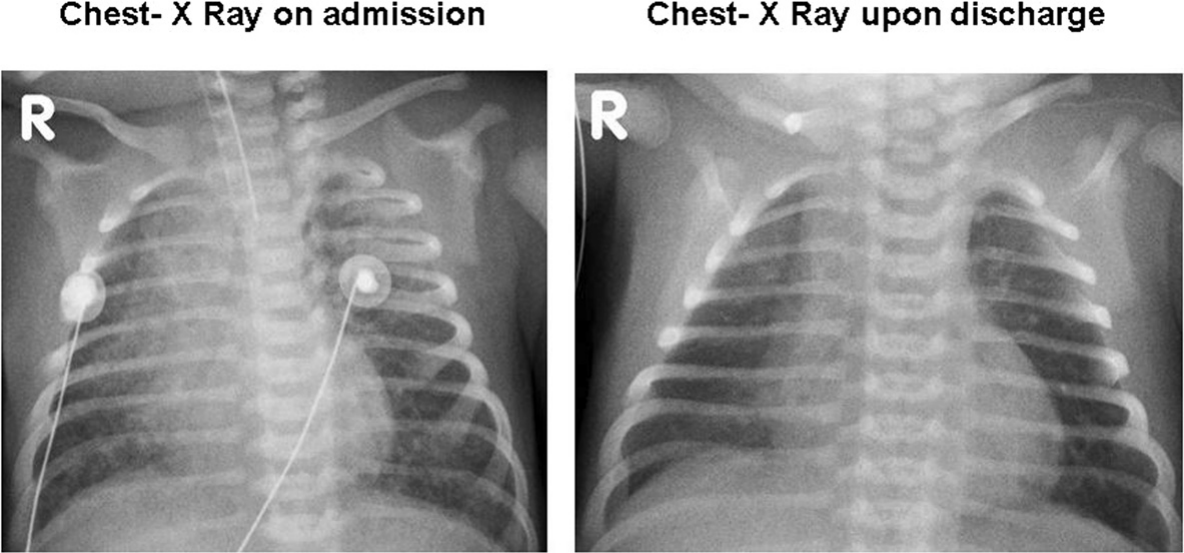
**Chest X-rays showing bilateral patchy infiltrates due to pulmonary hemorrhage on admission and resolution upon discharge.**

Upon arrival at the NICU, arterial blood gas analysis was normalized and routine investigations showed no signs of infection. Urea, creatinine and electrolytes were normal; liver transaminases were equally normal, as were alkaline phosphatase, bilirubin and albumin. The coagulation was severely disrupted with a prolonged prothrombin time and an international normalised ratio of 2.64. This was corrected by administration of fresh frozen plasma and an additional parenteral administration of Vitamin K. The patient received packed red cells to correct anaemia. Chest X-ray revealed bilateral patchy pulmonary infiltrates (Figure 1).

In the following hours, the patients’ respiratory state deteriorated. We observed a severe and progressive respiratory acidosis (pH 7.11, pO2 74.9 mmHg, pCO2 72.7 mmHg, bicarbonate 17.6 mmol/l, base excess −8.6 mmol/l, lactate 1.5 mmol/l). Chest X-ray revealed progression of the pulmonary infiltrates. Mechanical ventilation became increasingly difficult with a need for higher positive end-expiratory pressures (PEEP) and peak-inspiratory pressures during synchronized intermittent mandatory ventilation (SIMV). High frequency oscillation ventilation was tried, but SIMV with high PEEP (up to 10 cm H_2_0) proved most successful. Meanwhile, repetitive endotracheal suctioning continued to clear bloody secretions from the airways. The clinical presentation was that of a pulmonary haemorrhage. Echocardiography showed a normal size and shape of the heart as well as a normal cardiac function. Cranial ultrasound was normal. After stabilization, computed tomography of the chest was performed in order to identify any underlying intrathoracic lesion or vascular anomaly. Apart from massive pulmonary infiltrates, no structural lesions were found. Bronchoscopy could not identify obvious upper airway injuries. Since cardiac function was normal and no underlying lesion had been identified, the only clue to the origin of the pulmonary haemorrhage was the initial coagulopathy.

The coagulopathy was compatible with a disseminated intravascular coagulopathy since platelet count dropped from 234,000/μl (day 2) to 183,000/μl (day 5) (reference 140,000-440,000) and fibrinogen was at its nadir 99 mg/dl (day 2) (reference 160–415) but restored to 391 mg/dl at day 14. Laboratory signs of infection and blood cultures remained negative; these and other test results are shown in Table 1. In absence of other plausible explanations, a metabolic disorder was suspected and a first-line metabolic workup was executed during the acute phase. The patient quickly improved after his second day in hospital and mechanical ventilation was continued for 6 days, weaning was uncomplicated and on the seventh day of life, the boy was successfully extubated. Coagulation screens were repeatedly normal before discharge from hospital and no other bleedings were observed.

**Table 1:** **Laboratory data**

**Variable**	**At referral from other hospital, Day 2**	**On admission to this hospital, Day 2**	**Day 3**	**Day 12**	**Normal range**
C-reactive protein (mg/dl)	<0.1	<0.5	<0.5		<0.5
Haematocrit (%)	42.8	33	42		42 - 60
Haemoglobin (g/dl)	16.3	11.5	14.6		13,5 - 20,5
Mean corpuscular volume (μm3)	92	105.4	102.8		88 - 104
White-cell count (per mm3)	18.4	16.8	13.5		6.0 - 17.5
Platelet count (per mm3)	234000	220000	183000		140,000 - 440,000
Prothrombin time (%)		27	51	79	60 - 100
International normalised ratio		2.64	1.58	1.04	
Partial-thromboplastin time (sec)		131*	30	35	22 - 38
Fibrinogen (mg/dl)		99	113	391	160 - 415
Sodium (mmol/l)		143	134	139	132 - 146
Potassium (mmol/l)		4.67	4.2	4.63	3,5 - 5,0
Chloride (mmol/l)		106		103	94 - 110
Bicarbonate (mmol/l)		16		22	22 - 29
Calcium (mEq/l)		4.2		5.3	4.3 - 5.1
Glucose (mg/dl)	24	167	163	79	70 - 115
Creatinine (mg/dl)		0.85		0.39	0.24 - 0.85
Total bilirubin (mg/dl)		5.31		15.6	1.50 - 12.00
Protein (g/dl)		4.4	5	5.6	4.6 - 6.8
Albumin (g/dl)		3.5	3.6	4	2.8 - 4.4
Alanine aminotransferase (U/l)		29	34		<41
Aspartate aminotransferase (U/l)		59	88		<37
Lactate dehydrogenase (U/l)		1040			240 - 480
Creatine kinase (U/l)		1658			<652
**Blood gas analysis**					
pH	7.26	7.351	7.115		7.35 - 7.45
pO2 (mmHg)		147	74.9		83 - 108
pCO2 (mmHg)		33.2	72.7		35 - 48
Actual base excess (mmol/l)		-6.4	-8.6		-2.0 - +3.0
Lactate (mmol/l)		1.8	1.4		0.5 - 1.6

*Prolonged partial-thromboplastin time might have been the consequence of heparin in the umbilical catheter when the blood sample was taken. Control sample without heparin 5 hours later showed a partial-thromboplastin time of 34 seconds, a normal value; INR remained prolonged 1.80 and prothrombin time was 41%.

**Figure 2:**
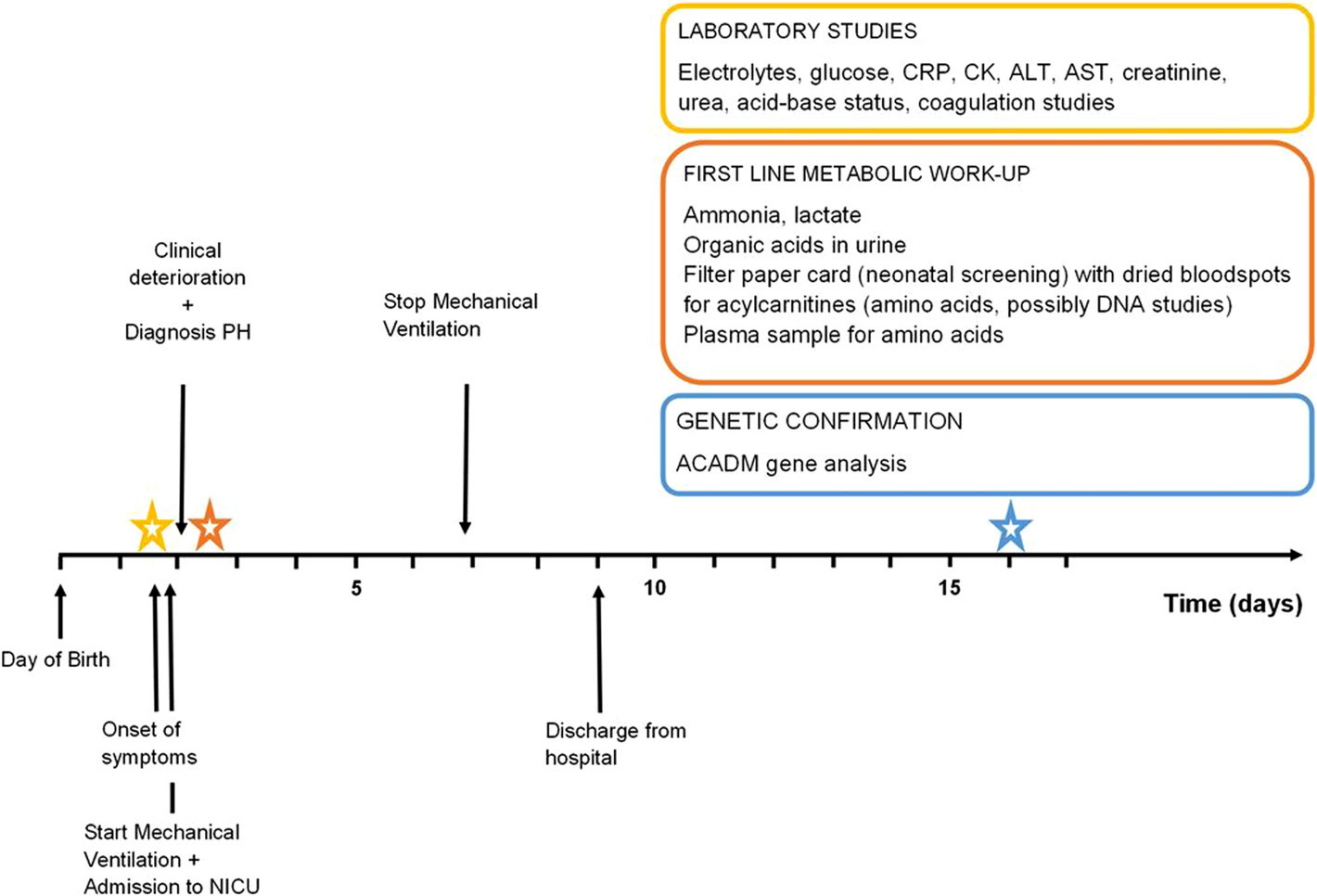
**Timeline showing work-up to diagnosis of MCADD.** ACADM: acyl-CoA dehydrogenase, C-4 to C-12 straight chain (MIM:607008), ALT: Alanine transaminase, AST: Aspartate transaminase, CK: Creatine kinase, CRP: C-reactive protein, PH: pulmonary haemorrhage.

Delayed delivery of the sample to the lab made results of the neonatal screening (Guthrie) and the metabolic review only available after 14 days (Table 2). The plasma amino acid profile was normal. Tandem mass spectrometry showed elevated levels of C6 (1.07 μmol/l, normal range 0.09-0.26), C8 (4.45 μmol/l, normal range 0.05-0.23) and C10:1 (1.41 μmol/l, normal range 0.04-0.25) acylcarnitines, consistent with Medium-chain Acyl-CoA dehydrogenase deficiency (MCADD) (#MIM 201450). A urinary organic acid profile collected on day 14 demonstrated a mild hypoketotic dicarboxylic aciduria with increased excretion of 5-hydroxyhexanoate (48 mmol/mol creat, reference <26) and octanoate (7 mmol/mol creat, reference <2), but absence of acylglycine accumulation. Urinary organic acid profile during decompensation was not available. Sequence analysis of the ACADM gene detected the common homozygous A985G missense mutation, confirming the diagnosis. Carnitine supplementation was initiated and avoidance of fasting through frequent feeding was advised. A schematic representation of the clinical course is reported in Figure 2. At the age of 36 months, the infant remains well with normal growth and development.

**Table 2:** **First line metabolic work-up**

**Variable**	**Day 14**	**Normal range**
Ammonia (μg/dl)	113	<228
Plasma amino acids		Normal
Urine organic acids		
Lactate (mmol/mol creat)	76	<79
3-Hydroxybutryrate (mmol/mol creat)	0	<26
Acetoacetate (mmol/mol creat)	0	<9
2-Oxoglutarate (mmol/mol creat)	942	<723
5-Hydroxyhexanoate (mmol/mol creat)	48	<26
Adipate (mmol/mol creat)	15	<30
Hexanoylglycine (mmol/mol creat)	0	<2
Octanoate (mmol/mol creat)	7	<2
Sebacate (mmol/mol creat)	20	<4
Suberate (mmol/mol creat)	20	<11
Suberglycine (mmol/mol creat)	0	<1
Acylcarnitine profile		
Free carnitine (μmol/l)	27.88	17.7 – 48.8
*Saturated acylcarnitines*		
Hexanoyl carnitine C6 (μmol/l)	1.07	0.09 - 0.26
Octanoyl carnitine C8 (μmol/l)	4.45	0.05 - 0.23
Decanoyl carnitine C10 (μmol/l)	0.48	0.06 - 0.31
*Unsaturated acylcarnitines*		
Decanoyl C10:1 (μmol/l)	1.41	0.04 – 0.25
Dodecanoyl C12:1 (μmol/l)	0.05	n.d. - 0.20

Table 2. Results from basic metabolic investigations.

**Interpretation urine organic acids**: Mild hypoketotic dicarboxyluria. Increased excretion of 5-hydroxyhexanoate and octanoate, absense of suspect glycine conjugates (suberylglycine and hexanoylglycine). Normal ratio of adipine, suberine and sebacine acid.

***Conclusion*** *: Mildly disturbed profile, not diagnostic. Differential diagnosis includes mild physiologic dicarboxylaciduria in children under 6 months, MCT diet or MCAD deficiency. To be correlated with clinical presentation and acylcarnitine profile.*

**Interpretation acylcarnitine profile:** Raised C6, C8, C10, C10:1 and C12:1.

***Conclusion:*** *Disturbed profile compatible with MCAD deficiency.*

## Discussion

Clinically significant pulmonary haemorrhage in newborns occurs at a rate of 1 to 12 per 1000 live births, and in term infants is usually associated with meconium aspiration, hypotension or resuscitation with positive pressure ventilation [1]. The exact pathogenesis of pulmonary haemorrhage remains unknown, although several theories have been suggested. In most cases of neonatal pulmonary haemorrhage, the lung effluent is thought to be haemorrhagic edema rather than blood [1]. Berger et al. speculate that in near-term or term infants suffering from intrauterine hypoxia, pulmonary hypertension and myocardial dysfunction might elevate pulmonary capillary pressure, causing stress failure and intra-alveolar haemorrhage [1].

Medium-chain Acyl-CoA dehydrogenase deficiency (MCADD) is the most common inherited disorder of mitochondrial fatty acid beta-oxidation. The overall frequency of the disorder is highest in northern Europe and has been estimated to range between 1:4,900 and 1:17,000 [2,3]. Fatty acid beta-oxidation fuels hepatic ketogenesis, a major source of energy once hepatic glycogen stores become depleted [3]. In a typical clinical scenario, a previously healthy child with MCADD presents between the third month and the second year of life with hypoketotic hypoglycaemia, vomiting, and lethargy, which can quickly progress to coma and death [3]. Acute episodes are generally triggered by a common illness or by prolonged fasting and liver disease is often seen at presentation. The prognosis of MCADD is excellent once the diagnosis is established and frequent feedings are instituted [3].

The patient presented in this case report probably developed hypoglycaemia and encephalopathy on the second day of life, resulting in inadequate feeding and further deterioration of his neurological condition. These initial signs of metabolic decompensation are often subtle and the rapidly progressive respiratory symptoms quickly dominated the clinical presentation, imposing the need for mechanical ventilation. Sepsis was the initial working diagnosis, but the absence of infectious signs together with a severe coagulopathy and a subsequent massive pulmonary haemorrhage raised suspicion towards an underlying metabolic disorder. So far, pulmonary haemorrhage in MCADD has only been reported in four post-mortem [5] and one surviving case [4]. In the latter, pulmonary haemorrhage was suggested to result from a transient, post-asphyxial cardiomyopathy, documented by cardiac ultrasound examination [4]. Although the clinical course was overall identical in our patient, cardiac function was repeatedly found to be normal. In contrast, both the patient presented by Maclean et al. and our patient were in an anticoagulated state. One obvious explanation could be liver dysfunction which is commonly seen in MCADD as a consequence of microvesicular hepatic steatosis due to the accumulation of medium chain fatty acids and secondary hepatocellular dysfunction [6]. However, transaminases were not elevated and the exact underlying mechanism of the coagulopathy remains uncertain. Indeed, the role of coagulopathy in pulmonary haemorrhage of any origin remains obscure, many studies have implicated activation of inflammation and derangement of the coagulation and fibrinolytic pathways in patients with acute lung injury or acute respiratory distress syndrome [7]. The clinical presentation with respiratory distress makes a spontaneous onset of the pulmonary haemorrhage triggered by metabolic decompensation probable. However, pulmonary haemorrhage may also have been aggravated by positive pressure ventilation during resuscitation.

## Conclusion

This is, to our knowledge, the second case reported in which a clinically significant pulmonary haemorrhage has been described in a surviving newborn infant with MCADD. In the only other surviving infant reported so far, pulmonary haemorrhage has been suggested to be a pre-terminal complication caused by heart failure. The key towards successful management of severe pulmonary haemorrhage in newborns with a coagulopathy and suspicion of an underlying metabolic disorder consists of adequate mechanical ventilation, aggressive use of fresh frozen plasma, while treating the metabolic decompensation by reversing catabolism and sustaining anabolism and initiating an early diagnostic work-up. We believe that in patients with MCADD, metabolic decompensation can cause a severe coagulopathy and a pulmonary haemorrhage, independent of cardiac function. Hence, pulmonary haemorrhage should not be considered a pre-terminal complication caused by heart failure, and rather than withdrawing care, intensive treatment must be initiated.

## Consent

Written informed consent was obtained from the patient for publication of this case report. A copy of the written consent is available for review by the Editor-in-Chief of this journal.

## Abbreviations

MCADD: Medium-chain Acyl-CoA dehydrogenase deficiency
NICU: Neonatal intensive care unit
PEEP: Positive end-expiratory pressure
SIMV: Synchronized intermittent mandatory ventilation
